# The efficacy of continuing aspirin in the perioperative period during percutaneous nephrolithotomy: A systematic review and meta-analysis

**DOI:** 10.12669/pjms.41.7.12240

**Published:** 2025-07

**Authors:** Lingling Hua, Hui Zhou

**Affiliations:** 1 Lingling Hua, Operating Room, Third People’s Hospital of Changxing County, Huzhou, Zhejiang Province 313103, P.R. China; 2 Hui Zhou, Department of Operating Room, Changxing County People’s Hospital, Huzhou, Zhejiang Province 313100, P.R. China

**Keywords:** Urolithiasis, Kidney stones, Anti-platelet drugs, Acetylsalicylic acid

## Abstract

**Objective:**

The present study was conducted to assess the safety of continuing aspirin during percutaneous nephrolithotomy (PCNL).

**Methods:**

PubMed, CENTRAL, Scopus, Embase, and Web of Science were searched for relevant studies up to 5^th^ February 2025. Random-effects meta-analysis was conducted for change in hemoglobin, blood loss, length of hospital stay, complications, need for transfusion and postoperative thrombotic events between patients continuing aspirin vs patients not on any antithrombotic therapy in the perioperative period (controls).

**Results:**

Six studies were included. Meta-analysis showed no statistically significant difference in change in hemoglobin levels (MD: -0.03 95% CI: -0.24, 0.18 I^2^=31%), estimated blood loss (MD: -6.91 95% CI: -14.36, 0.54 I^2^=0%), length of hospital stay (MD: -0.31 95% CI: -0.99, 0.37 I^2^=94%), all complications (OR: 1.29 95% CI: 0.94, 1.79 I^2^=0%), serious complications (OR: 1.95 95% CI: 0.88, 4.29 I^2^=38%), bleeding complications (OR: 1.11 95% CI: 0.71, 1.73 I^2^=0%), need for transfusion (OR: 1.10 95% CI: 0.62, 1.94 I^2^=0%), and postoperative thrombotic events (OR: 1.30 95% CI: 0.21, 8.24 I^2^=36%) between patients continuing aspirin and controls.

**Conclusions:**

Continuing aspirin during the perioperative period may not increase the risk of adverse outcomes of PCNL. However, given the scarce data further prospective and Multi-Centre studies are needed to improve the quality of evidence.

## INTRODUCTION

Urinary tract stone or urolithiasis is one of the most common urological ailments that has a prevalence of 0.1-14.8% in Western populations and about 10.6% in Asians.^1^,^2^ It is also a common cause of pain seen in the emergency department often presenting with significant flank pain, infection, hydronephrosis, and reduced renal function.^3^,^4^ Asymptomatic stones can be managed with observation but symptomatic cases often require treatment with expulsive therapy, extracorporeal shockwave lithotripsy, percutaneous nephrolithotomy (PCNL), or retrograde intrarenal surgery.^5^-^7^

PCNL is often used for complex large renal calculi especially >20mm in size. It can also be a substitute to retrograde intrarenal surgery for 10-20mm stones due to better success rates.^8^ However, a few contraindications of PCNL include pregnancy, bleeding disorders or under anticoagulation, uncontrolled urinary tract infections, and tumors in the presumptive access tract area.^8^,^9^ The concepts have evolved with time leading to smaller equipment and advancements in energy so that even smaller stones can be managed with PCNL resulting in reduced adverse events and high stone clearance rates.^7^,^8^ With increasing life expectancy, the elderly now constitute a significant number of patients presenting with renal calculi.^10^ Such patients usually have comorbidities and are under various medications which makes management complicated. Geriatric joint-related, cardiovascular, and cerebrovascular diseases and their treatments often require prolonged antiplatelet or anticoagulant drugs to reduce the risk of systemic thromboembolism.^11^,^12^

However, the use of antiplatelets in the perioperative period can be a risk factor for bleeding during surgery.^13^ This is a cause of concern in PCNL as it is categorized as a high-risk procedure for bleeding and the European Association of Urology recommends discontinuation of aspirin before the procedure.^14^ Nevertheless, there are reports which indicate that PCNL can be safely performed without discontinuation of aspirin as stoppage of the same can lead to rebound thrombosis and risk of cardiovascular events.^15^ A prior meta-analysis of Pan et al^15^ has pooled data from four studies to show that there may be no difference in the risk of bleeding and perioperative complications between patients on continued aspirin therapy vs controls. Given the small number of studies included in their review, there is a need for more updated evidence. We, therefore, investigated the safety of performing PCNL without discontinuation of aspirin by this systematic review and meta-analysis.

## METHODS

### Inclusion criteria:

We conducted this PRISMA-compliant^16^ (Supplementary file) and PROSPERO-registered (CRD42025645404) study to examine the effects of continuing aspirin during PCNL. The eligibility criteria were formulated by all reviewers by consensus to include all observational study designs conducted on PCNL patients. Studies compared outcomes of patients continuing aspirin therapy during the procedure with controls. Controls were defined as patients not on any antithrombotic therapy during the perioperative period. Outcomes to be reported were either of the following: bleeding complication, blood loss, change in hemoglobin, need for transfusion, overall complications, thrombotic events, or length of hospital stay (LOS).

### Exclusion Criteria:

Studies on mixed interventions and not reporting separate outcomes for PCNL were excluded. We also excluded articles available only as abstracts and in non-peer-reviewed journals.

### Search strategy:

A search query was devised by two reviewers for all literature databases, which was: (antiplatelet) OR (antithrombotic)) OR (aspirin)) OR (acetylsalicylic acid) OR (blood thinner)) OR (platelet inhibitor)) AND (Percutaneous nephrolithotomy) OR (PCNL)). This query was utilized on the websites of PubMed, CENTRAL, Scopus, Embase, and Web of Science (Supplementary Table-I). We finished the search on 5^th^ February 2025. No restriction was placed on language.

**Supplementary Table I:** Search strategy.

Embase:
1. ‘antiplatelet’/exp OR ‘antiplatelet’
2. anti-thrombotic
3. ‘aspirin’
4. ‘acetylsalicylic acid’
5. ‘platelet inhibitor’
6. ‘blood thinner’
7. #1 OR #2 OR #3 OR #4 OR #5 OR #6
8. ‘Percutaneous nephrolithotomy’
9. ‘PCNL’
10. #8 OR #9
11. #7and #10
***PubMed:*** (antiplatelet) OR (antithrombotic)) OR (aspirin)) OR (acetylsalicylic acid) OR (blood thinner)) OR (platelet inhibitor)) AND (Percutaneous nephrolithotomy) OR (PCNL))
Scopus: (TITLE-ABS-KEY-AUTH (antiplatelet OR antithrombotic OR aspirin OR acetylsalicylic acid OR blood thinner OR platelet inhibitor)) AND (TITLE-ABS-KEY-AUTH (Percutaneous nephrolithotomy OR PCNL))
***Web of Science:*** (antiplatelet) OR (antithrombotic)) OR (aspirin)) OR (acetylsalicylic acid) OR (blood thinner)) OR (platelet inhibitor)) AND (Percutaneous nephrolithotomy) OR (PCNL))
***CENTRAL:*** [(antiplatelet):ti,ab,kw OR (“antithrombotic”):ti,ab,kw OR (“aspirin “):ti,ab,kw OR (acetylsalicylic acid):ti,ab,kw OR (“blood thinner”):ti,ab,kw OR (platelet inhibitor):ti,ab,kw] AND (Percutaneous nephrolithotomy):ti,ab,kw OR (PCNL):ti,ab,kw].

The articles searched were combined and deduplicated. Studies were screened by title and abstracts and found relevant by either reviewer underwent further screening by full-text reading. Two reviewers separately conducted the final screening and all disagreements were resolved by consensus. We then also searched the reference lists of prior reviews and included studies.

### Data extraction:

Two authors extracted data from the studies independently of each other by using a pre-formulated table. All disagreements were resolved by consensus. Information on the study authors, place of the study, study design, sample size, age and gender of participants, mean stone size, body mass index (BMI), operative time, and study outcomes. Outcomes analyzed were change in hemoglobin, estimated blood loss, LOS, all complications, serious complications (defined as Clavien-Dindo Grade-III a or higher complications), bleeding complications, need for transfusion, and postoperative thrombotic events.

### Risk of bias:

All included articles were judged for bias in the methodology by two reviewers using the Newcastle-Ottawa Scale (NOS).^17^ The questions of the NOS cover three major domains namely, selection of the sample, comparability between groups, and the outcome assessment with studies marked with a score of zero to nine. Higher score meant better quality. All disagreements were resolved by consensus.

### Statistical analysis:

The statistical analysis was conducted on “Review Manager” (RevMan, version 5.3; Nordic Cochrane Centre (Cochrane Collaboration), Copenhagen, Denmark; 2014). Categorical or binary data was pooled to generate odds ratio (OR) and 95% confidence intervals (CI) while continuous data was combined to estimate the mean difference (MD). A random-effect meta-analysis model was used. Publication bias was examined using Egger’s test. Likewise, due to scarce data, subgroup analysis was also not possible. The I^2^ statistic provided the numerical value on heterogeneity between studies with I^2^>50% indicating substantial heterogeneity. Certainty of evidence was assessed using GRADE.

## RESULTS

**Fig.1:**
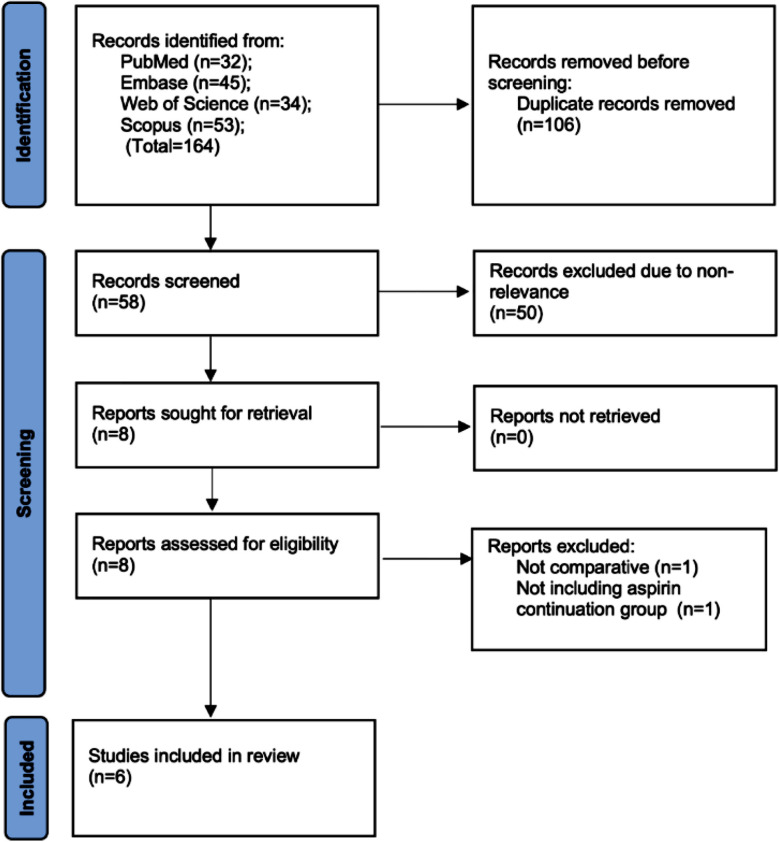
Flowchart of the review.

The PRISMA flowchart of the study demonstrated articles available at each step of the screening process (Fig.1). We retrieved 164 articles from the databases and were able to cut down to 58 studies after electronic deduplication. The 58 studies were meticulously examined by the reviewers by reading the title and abstracts and eight were selected for full-text review. Of these, six articles^18^-^23^ were included. Data from one study^18^ was included from the prior review.^24^ There was no inter-reviewer conflict in the inclusion of studies.

### Details of studies:

Characteristics of studies can be found in Table-I. There were four studies^19^,^20^,^22^,^23^ from the USA and one each from China^18^ and Iran.^21^ All were cross-sectional in design. Three hundred and six patients in the studies were in the experimental group continuing aspirin during the procedure while 1196 patients served as controls. The mean/median age of the patients was above 50 years in all studies. The studies did not note any significant difference in BMI of the two groups. Moreover, there was no significant difference reported in the stone size or volume noted between the groups. Operative time varied between 27 mins to 190 mins in the study groups. However, the studies reported no statistically significant differences in the operative time between the groups. The dose of aspirin was up to 100mg/day in most studies. Two reviewers awarded every study with a score of seven on NOS (Supplementary Table-II). Two points were deducted from each study as none matched the two groups on baseline characteristics. Therefore, there were no points for comparability.

**Table-I:** Information collected from the studies.

Study	Location	Design	Groups	Sample size	Age (years)	Males	BMI	Stone size (mm)	Operative time (mins)
Leavitt 2014^22^	USA	CS	CA Control	15 38	69 62	11 19	31.1* 32.9	21± 11 23± 14	74* 77
Otto 2017^23^	USA	CS	CA Control	67 207	66 52	37 100	32.1± 9 30.3±9	37± 16 40± 19	163± 62 190± 67
Wang 2019^18^	China	CS	CA Control	44 40	58.7 50.4	NR	NR	20.6± 5.2 21.3± 5	28.3± 7.1 27± 5.1
Falahatkar 2017^21^	Iran	CS	CA Control	40 603	60.1 48.7	16 331	28.6± 4.9 27.9± 4.9	32.9± 16.4 33.3± 13.2	43.2± 21.4 44.8± 16.8
Rosenbluth 2023^20^	USA	CS	CA Control	55 68	67.8 65.2	29 37	28.5± 7.7 29.8± 6.6	2058± 2092.7^ 796.9± 950.7	97.2± 58.2 100.4± 43.5
Agarwal-Patel 2023^19^	USA	CS	CA Control	85 240	65 66	49 137	30* 33	9± 5 8± 5	84* 86

CS, cross-sectional; CA, continued aspirin; NR, not reported; BMI, body mass index; ^stone volume,

* Median values.

**Supplementary Table II:** Risk of bias analysis.

Study	Selection of cohort	Comparability	Outcome assessment	NOS score
Leavitt 2014^22^	4	-	3	7
Otto 2017^23^	4	-	3	7
Wang 2019^18^	4	-	3	7
Falahatkar 2017^21^	4	-	3	7
Rosenbluth 2023^20^	4	-	3	7
Agarwal-Patel 2023^19^	4	-	3	7

NOS, Newcastle Ottawa scale.

### Meta-analysis:

**Fig.2:**
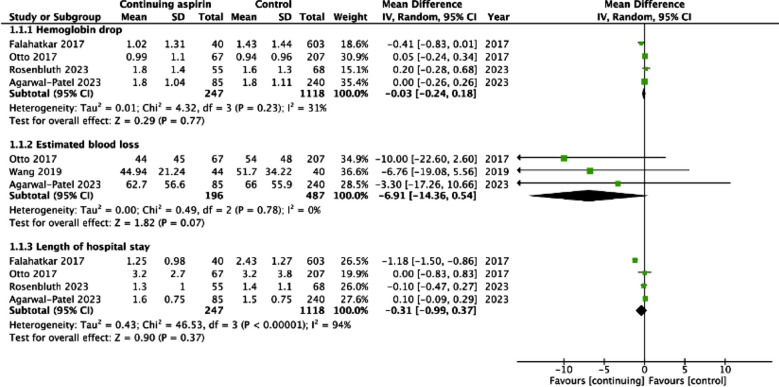
Meta-analysis of change in hemoglobin, estimated blood loss and length of hospital stay with continued use of aspirin during PCNL.

**Fig.3:**
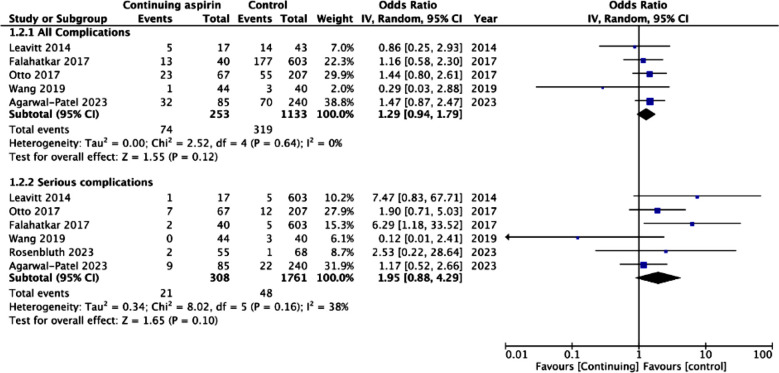
Meta-analysis of all complications and serious complications with continued use of aspirin during PCNL.

**Fig.4:**
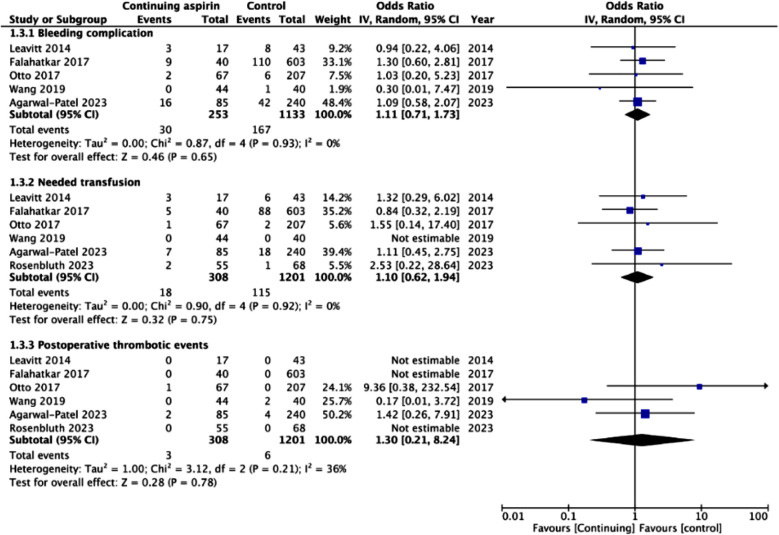
Meta-analysis of bleeding complications, need for transfusion and postoperative thrombotic events with continued use of aspirin during PCNL.

The meta-analysis showed no statistically significant difference in change in hemoglobin levels after the procedure between the two groups (MD: -0.03 95% CI: -0.24, 0.18 I^2^=31%) (Fig.2). No publication bias was noted on Egger’s test (p=0.54). We also noted no statistically significant difference in estimated blood loss (MD: -6.91 95% CI: -14.36, 0.54 I^2^=0%) and LOS (MD: -0.31 95% CI: -0.99, 0.37 I^2^=94%) between the two groups (Fig.2). No publication bias was noted on Egger’s test (p>0.05). Meta-analysis showed no significant difference in the risk of all complications (OR: 1.29 95% CI: 0.94, 1.79 I^2^=0%) and serious complications (OR: 1.95 95% CI: 0.88, 4.29 I^2^=38%) between the two groups (Fig.3). Risk of bleeding complications (OR: 1.11 95% CI: 0.71, 1.73 I^2^=0%), need for transfusion (OR: 1.10 95% CI: 0.62, 1.94 I^2^=0%), and postoperative thrombotic events (OR: 1.30 95% CI: 0.21, 8.24 I^2^=36%) did not differ between the two groups (Fig.4). None of the outcomes showed publication bias on Egger’s test (p>0.05). Certainty of evidence for all outcomes was ‘very low’ (Supplementary Table-III).

**Supplementary Table-III:** GRADE assessment of evidence.

	Change in hemoglobin levels	Estimated blood loss	Length of hospital stay	All complications	Serious complications	Bleeding complications	Need for transfusion	Postoperative thrombotic events
Number of studies	4	3	4	5	6	5	6	6
***Downgrade quality of evidence***								
Risk of bias	Serious^	Serious^	Serious^	Serious^	Serious^	Serious^	Serious^	Serious^
Inconsistency	No	No	No	No	No	No	No	No
Indirectness	No	No	No	No	No	No	No	No
Imprecision	No	No	No	No	No	No	No	No
Publication bias	No	No	No	No	No	No	No	No
***Upgrade quality of evidence***								
Large effect	No	No	No	No	No	No	No	No
Plausible confounding	No	No	No	No	No	No	No	No
Dose-response	No	No	No	No	No	No	No	No
Overall certainty of Evidence	Very low	Very low	Very low	Very low	Very low	Very low	Very low	Very low

^NOS score of studies was 7.

## DISCUSSION

Continuation of aspirin in the perioperative period has been a topic of controversy.^24^ The current study investigated the effects of continuing aspirin on intra-operative and post-operative outcomes of PCNL. Pooled data from six cross-sectional studies showed that continuation of aspirin may not result in excessive bleeding during the procedure. This was measured by several outcomes, namely, change in hemoglobin, estimated blood loss, bleeding complications, and need for transfusion. None of these outcomes showed statistically significant results. There seemed to be a tendency of greater blood loss in the aspirin group with a MD of 6.9ml, but the 95% CI remained non-significant. Examination of other important outcomes, namely, complications and serious complications also showed no differences between the study groups, indicating that the use of aspirin in the peri-operative period did not seem to increase the risk of all or serious complications.

These results are in congruence with those reported in the prior meta-analysis of Pan et al^15^ which collated data from four studies. The current study presented updated and more comprehensive evidence since we were able to add two more studies to their review and also conduct a meta-analysis on the change in hemoglobin which was missing in their meta-analysis. Moreover, the results also concur with other research in the urological literature. Ito et al^25^ have shown that continuation of aspirin during partial nephrectomy may not elevate the risk of bleeding. A meta-analysis also shows that the use of aspirin may have a limited effect on bleeding risk during kidney biopsy.^26^ Similarly, continued use of aspirin does not seem to increase surgical morbidity, blood loss, or LOS in patients undergoing robot-assisted radical prostatectomy.^27^ Wu et al^28^ have also shown that bipolar plasma-kinetic transurethral resection of the prostate seems to be safe in patients taking aspirin without increasing complication rates.

An important concern with the stoppage of aspirin preoperatively is the risk of rebound thrombosis due to aspirin withdrawal syndrome.^24^ Sudden stoppage of the drug can increase the thromboxane A2 activity and reduce fibrinolysis thereby accelerating platelet adhesion and aggregation. Another aggravating factor is the surgical trauma itself which causes a prothrombotic and proinflammatory state further accentuating platelet aggregation.^24^,^27^ A pooled analysis shows that discontinuation of aspirin significantly increases the risk of myocardial infarction and death with the event occurring after a mean of 10.6 days post-drug cessation.^29^ In the present study, the risk of thrombotic complications was not different between the two groups probably due to limited number of studies in the analysis.

A major drawback of the included literature was the lack of baseline matching of study and control groups with none conducting a propensity score-matched analysis. Several risk factors exist for bleeding after PCNL like diabetes mellitus, urinary tract infection, stone size, staghorn stone, procedural duration, degree of hydronephrosis, multiple tracts, and surgeon experience.^30^,^31^ The PCNL technique itself may influence bleeding rates of which the size of percutaneous renal access is deemed important. Smaller tract prevents trauma to the parenchymal tissue and the renal pelvis thereby reducing PCNL complication rates.^5^, The mini and ultra-mini PCNL is associated with significantly reduced blood loss, change in hemoglobin, need for transfusion, and LOS as compared to standard PCNL.^32^ Given the plethora of factors affecting bleeding risk with PCNL and the lack of adjusted data in the literature, future studies should take into account the risk factors of PCNL bleeding while reporting the effects of antithrombotic drugs.

### Limitations:

There were only six studies available for the meta-analysis which limits the statistical power. Not all studies reported complete outcomes and hence in some meta-analyses, the number of studies was further reduced. The study designs were retrospective and cross-sectional which increases the risk of selection bias. The decision of continuation of aspirin was taken by the clinicians and it is possible that patients with high risk of bleeding could have been preferentially asked to discontinue the drug. All studies had short follow-ups and evaluated only peri-operative outcomes. It is possible that bleeding or thromboembolic events may have been missed due to reduced follow-up. Data included in the review was also mostly from the USA. PCNL practices and peri-operative protocols differ by region and hence generalizability of the present results may be limited.

Present guidelines still suggest that aspirin should be discontinued before PCNL and the patient’s primary physician should be consulted before discontinuing the drug.^14^ While the current results demonstrate that continuation of aspirin may not increase the risk of bleeding and other adverse outcomes, the quality of evidence is low to make definitive recommendations. The several limitations outlined above do not permit a change in present guidelines till further high-quality evidence preferably from randomized controlled trials is made available. Till then, we recommend that the decision to continue aspirin should be based on the individual patient risk factors and disease severity in consultation with the primary physician who started the anti-thrombotic therapy. Patients should be informed of the risks and benefits of continuing aspirin during the procedure before the final decision is made. We also recommend that robust multicentric randomized controlled trials with long follow-up should be conducted with a common PCNL and peri-operative protocol to generate high quality evidence.

## CONCLUSIONS

Continued use of aspirin during PCNL may not increase the risk of bleeding and complications. Meta-analysis shows that there may not be any difference in change in hemoglobin levels, estimated blood loss, length of hospital stay, all complications, serious complications, bleeding complications, need for transfusion, and postoperative thrombotic events. Further high-quality studies are needed to validate the present findings.

### Authors’ contributions:

**LH:** Literature search, study design and manuscript writing. Revision and validation and is responsible for the integrity of the study.

**LH and HZ:** Data collection, data analysis and interpretation. Critical Review.

All authors have read and approved the final manuscript.
